# Comment on “Microsurgical Techniques Used to Construct the Vascularized and Neurotized Tissue Engineered Bone”

**DOI:** 10.1155/2015/685412

**Published:** 2015-05-06

**Authors:** Annika Weigand, Justus P. Beier, Andreas Arkudas, Raymund E. Horch, Anja M. Boos

**Affiliations:** Department of Plastic and Hand Surgery, University Hospital of Erlangen, Friedrich-Alexander University of Erlangen-Nürnberg, Krankenhausstraße 12, 91054 Erlangen, Germany

We read with great interest the review article by Fan et al. [1] that discusses a range of microsurgical techniques used for vascularization and neurotization in bone tissue engineering. Not only the lack of vascularization but also the lack of neurotization within tissue engineered bone can lead to insufficient ossification resulting in treatment failure.

Since we have been working in the research area of vascularized bone tissue engineering for many years, we are aware of the problem of generating an optimal prevascularized scaffold for large and poorly vascularized bone defects. One of the main obstacles remains an adequate vascularization and thus survival of implanted cells or, with regard to the manuscript by Fan et al., of nerve tracts, especially in the center of scaffolds.

As mentioned by Fan et al. in a range of studies it could be demonstrated that sensory nerve innervation can contribute to bone integrity and bone formation [2–4]. Chen et al. demonstrate that sensory nerves could enhance the osteogenesis of tissue engineered bone. Sensory nerves or blood vessels were implanted with a scaffold directly in the defect site [3]. Fan et al. emphasize that an optimal support with oxygen and nutrients is of fundamental importance for the survival of the neurocytes. For optimization and constructing the vascularized and neurotized tissue engineered bone simultaneously both blood vessel and the nerve tract should be implanted together in the bone graft [1].

Another way for engineering vascularized tissue is the prevascularization of constructs distant to the injury site in unharmed areas of the body using the intrinsic vascularization with an arteriovenous (AV) loop that was firstly described by Erol and Spira [5]. After a sufficient prevascularization period osteogenic cells such as osteoblasts or nerve tracts can be implanted without being lost through inadequate supply of oxygen or nutrients. Tissue engineered constructs can afterwards be microsurgically transplanted in the defect site independent of the local vasculature. In a previously performed study in the rat AV loop model space holders were placed in the scaffold during the prevascularization period, leaving holes for subsequent injection of CFDA-labeled osteoblasts. A significantly higher number of CFDA-positive cells and a higher expression of bone specific markers were detected in the prevascularized scaffolds compared to control, indicating this technique to be well suited to support bone formation and the probable survival of implanted cells.

Nevertheless, moving from bench to bedside, the large animal sheep AV loop model was established, and we were able to generate intrinsically vascularized tissue in a clinically relevant size (volume 15 cm³). For faster vascularization and consequently better nutritional support of implanted cells, we modified the AV loop model using a perforated chamber. Combining extrinsic and intrinsic vascularization, enhancement of angiogenesis could be demonstrated in the small and large animal [6] (Figure 1). Connection between both vascular pathways allows the microsurgical transplantation of the vascularized bone construct. Based on the results of our studies, we have previously used the AV loop successfully for the reconstruction of large bone defects in two patients [7].

To pave the way for further preclinical testing, the efficient implantation of a sensor nerve tract by Fan et al. should be proofed in a large animal and combined with the intrinsic-extrinsic large animal AV loop model. For minimizing hypoxic-dependent cell death, the nerve tract could be implanted after an adequate prevascularization period or close to AV loop vessels. We agree with Fan et al. that further studies must be performed to find efficient therapies for large and complicated bone defects, in order to make wide clinical application possible.

## Figures and Tables

**Figure 1:**
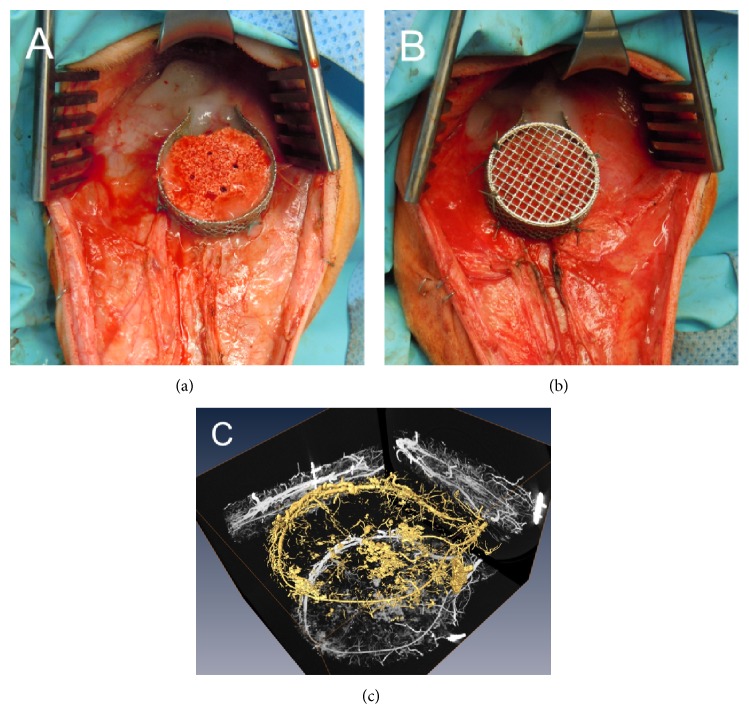
Arteriovenous loop model sheep. ((a)-(b)) Implantation of a primary stable bone substitute within a perforated titanium chamber allowing combined intrinsic-extrinsic vascularization of the scaffold. (c) After explantation vessels were perfused with a contrast agent and visualized using micro-CT.
